# Visualizing Knowledge Evolution Trends and Research Hotspots of Personal Health Data Research: Bibliometric Analysis

**DOI:** 10.2196/31142

**Published:** 2021-11-01

**Authors:** Jianxia Gong, Vikrant Sihag, Qingxia Kong, Lindu Zhao

**Affiliations:** 1 School of Economics and Management Southeast University Nanjing China; 2 Department of Industrial Engineering and Innovation Sciences Eindhoven University of Technology Eindhoven Netherlands; 3 Department of Technology and Operations Management Erasmus University Rotterdam Rotterdam Netherlands

**Keywords:** knowledge evolution trends, research hotspots, personal health data, bibliometrics

## Abstract

**Background:**

The recent surge in clinical and nonclinical health-related data has been accompanied by a concomitant increase in personal health data (PHD) research across multiple disciplines such as medicine, computer science, and management. There is now a need to synthesize the dynamic knowledge of PHD in various disciplines to spot potential research hotspots.

**Objective:**

The aim of this study was to reveal the knowledge evolutionary trends in PHD and detect potential research hotspots using bibliometric analysis.

**Methods:**

We collected 8281 articles published between 2009 and 2018 from the Web of Science database. The knowledge evolution analysis (KEA) framework was used to analyze the evolution of PHD research. The KEA framework is a bibliometric approach that is based on 3 knowledge networks: reference co-citation, keyword co-occurrence, and discipline co-occurrence.

**Results:**

The findings show that the focus of PHD research has evolved from medicine centric to technology centric to human centric since 2009. The most active PHD knowledge cluster is developing knowledge resources and allocating scarce resources. The field of computer science, especially the topic of artificial intelligence (AI), has been the focal point of recent empirical studies on PHD. Topics related to psychology and human factors (eg, attitude, satisfaction, education) are also receiving more attention.

**Conclusions:**

Our analysis shows that PHD research has the potential to provide value-based health care in the future. All stakeholders should be educated about AI technology to promote value generation through PHD. Moreover, technology developers and health care institutions should consider human factors to facilitate the effective adoption of PHD-related technology. These findings indicate opportunities for interdisciplinary cooperation in several PHD research areas: (1) AI applications for PHD; (2) regulatory issues and governance of PHD; (3) education of all stakeholders about AI technology; and (4) value-based health care including “allocative value,” “technology value,” and “personalized value.”

## Introduction

Over the past 20 years, the use of patient medical information has rapidly increased in both clinical practice and research [1,2]. Improved access to personal health data (PHD), thanks to emerging technologies such as wearable devices, and mobile phones have improved health care delivery and physician–patient relationships, particularly for patients with noncommunicable chronic diseases [3]. PHD can play an important role in providing patient-centered rather than disease-centered health care by facilitating health care providers to learn about an individual’s medical history and current health status [4-6]. At the same time, this data-driven approach is helping to provide cost-effective and high-quality health care—known as value-based health care [7]. It is expected that PHD will continue to transform the health care industry.

**Figure 1:**
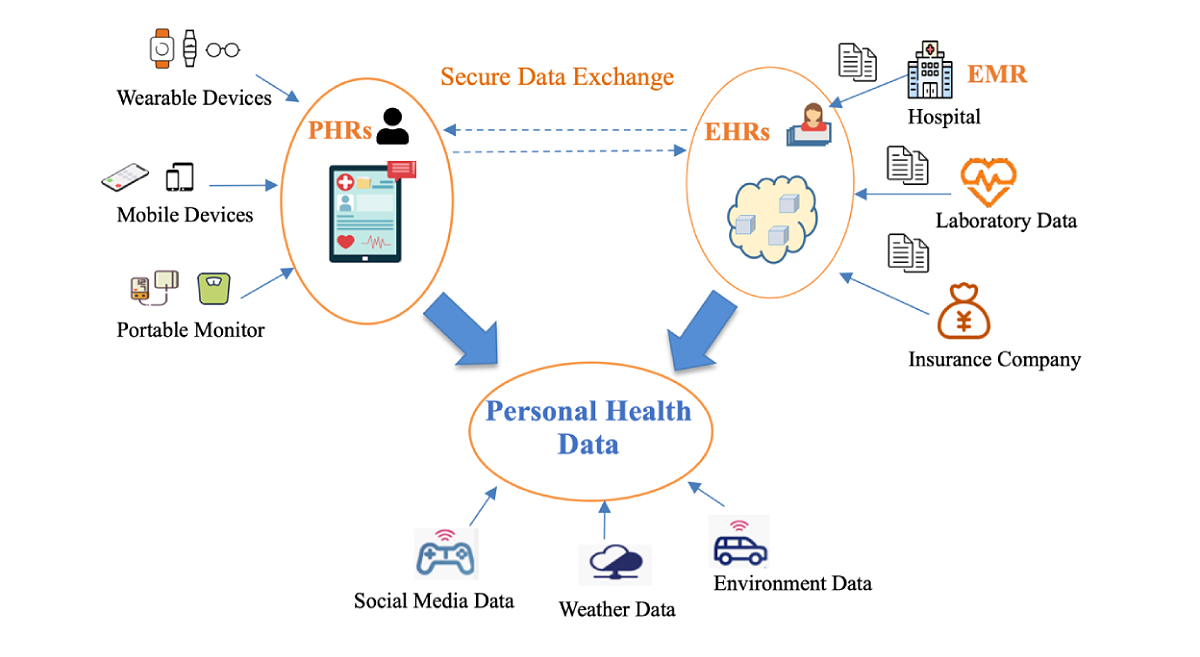
PHR, EHR, and EMR relationships. EHR: electronic health record; EMR: electronic medical record; PHR: personal health record.

PHD includes both clinical data (eg, electronic medical records [EMRs], electronic health records [EHRs], personal health records [PHRs]) and nonclinical data (eg, sentiments, emotions, characteristics, and social media behavior) [2]. Figure 1 shows the relationship between EMR, EHR, PHR, and PHD. EMR files are real-time electronic files including only clinical records that have replaced paper files; these are usually not sent to other health care providers outside the treating hospital or clinic [8]. This transition to electronic records signifies a great digital transition in the health care industry. The standardization of EHR has provided a repository of health information that has greatly facilitated interoperability between different institutions [2]. EHR usually belongs to health care organizations [9] and cannot be easily transmitted between different organizations because of different data standards and health information systems. To overcome this limitation, PHR was generated [6]. PHRs are electronic records of health-related information that conform to national interoperability standards and can be drawn from multiple sources (eg, EHRs, laboratory test results, smartphones, and wearable devices), while being managed, shared, and controlled by the individual [10].

Health care providers now have access to clinical data from EHR and patients’ self-reported health data (eg, test results, medication lists, allergies) from PHRs. However, they do not have access to the patients’ self-reported experiences, attitudes, feelings, and emotional states. The development of the internet of things and wearable devices means that PHD can also include nonclinical health-related data, such as daily physical activity and diets. Individuals are now sharing more and more detailed health information via social media platforms such as Twitter and through online health communities such as PatientsLikeMe [11]. Hill [12] defined PHD as any data related to an individual’s health condition [12], while Plastiras and O’Sullivan [13] viewed PHD as health data generated by patients during their daily life. In this study, PHD is defined as data related to clinical and nonclinical well-being, including EMR, EHR, PHR, and environment and social media data. Incorporating broader nonclinical PHD such as emotions and feelings has been shown to enhance personalized health care delivery [14,15].

PHD research has gained attention in various fields, including computer science, bioinformatics, medicine, and public health. Searching for the keyword “personal health data” on Web of Science shows that relevant articles on PHD have increased greatly (Multimedia Appendix 1). Several systematic reviews have been published on different topics associated with PHD (Table 1). These include security and privacy problems associated with EHR [16], data types and standardization [6], facilitators and barriers to using EHR in the United States [17,18], barriers to data sharing [19], and ethical issues of data collection [20]. Others have investigated factors affecting the use of PHR and big data applications of PHD [11,21,22]. While the PHD research literature grows rapidly, some scholars acknowledged the value of presenting comprehensive landscape and topic evolution process of PHD publications for researchers in various disciplines, in which bibliometric as a quantitative analysis method can be useful. Some scholars analyzed the status and detected the high-frequency terms of EHR [23-26]. Wen et al [27] analyzed the production trends of publications on EHRs by countries from 2009 to 2015. Wang et al [28] used bibliometric methods to compare publication hotspots in EHRs from different periods among 6 countries. The recent articles by Qian et al [29] and Zhenni and Yuxing [30] applied social network analysis and topic modeling methods to explore the EHR publications in-depth to evaluate the publications trends and detect the frontiers. However, these were mainly aimed at a specific type of health data: EHR. Karampela et al [2] used a systematic mapping approach to present the publication channel, publication year, and major research topics to provide a more complete overview of PHD research. However, it is not clear what phase each topic is in, how each topic is progressing, what knowledge trends are evolving, and which topics will become research hotspots.

This study aims to examine the evolving trends and to detect the potential research hotspots of PHD by identifying, classifying, and clustering PHD research topics from 2009 to 2018. We used knowledge evolution analysis (KEA) with bibliometric techniques to review articles retrieved from the Web of Science database. This study traces the evolution of PHD using knowledge networks based on reference co-citation, keyword co-occurrence, and discipline co-occurrence. Revealing the interrelationships between PHD research topics will provide a solid framework for future research. Table 2 presents the key questions that will be answered in this study.

**Table 1:** Comparison of literature reviews.

Study	Research question	Sample size	Time range	Method
Archer et al [17]	PHRs^a^ design, functionality, implementation, application, outcomes, and benefits	130	Unlimited-2010	Systematic review
Fernández-Alemán et al [16]	Security and privacy in EHRs^b^	49	2006-2011	Systematic review
Van Panhuis et al [19]	Barriers to data sharing	65	Unlimited-2013	Systematic review
Kruse et al [18]	Adoption factors of EHRs	31	2012-2015	Systematic review
Roehrs et al [6]	Data types, standards, profiles, goals, methods, functions, and architecture with PHRs	97	2008-2017	Systematic review
Yin et al [11]	Machine learning in online personal health data	103	2010-2018	Systematic review
Maher et al [20]	Ethical issues in passive data collection	48	Unlimited-2018	Systematic review
Abd-alrazaq et al [21]	Factors affecting the use of PHRs	97	2000-2018	Systematic review
Mehta and Pandit [22]	Big data analytics in PHD^c^	58	2013-2018	Systematic review
Wang et al [28]	Evolution of publication hotspots in EHRs	17,678	1957-2016	Bibliometric method
Wen et al [27]	Production trends of EHR	1803	1991-2005	Bibliometric method
Guo et al [23]	Status, hotspots of EHR	5095	2005-2010	Bibliometric method
Liang et al [24]	Status, directions of EHR	1262	1990-2013	Bibliometric method
Ruixian et al [25]	Status of EMR^d^ in China	262	1999-2004	Bibliometric method
Zhenni and Yuxing [30]	Hot spots in EHR	13,438	1900-2019	Bibliometric method
Qian et al [29]	Landscape, hot topics, trends of EHRs	13,438	1900-2019	Bibliometric method
Lin et al [26]	Status of EMR research in China	1752	1999-2012	Bibliometric method
Karampela et al [2]	Publication source, publication year, research topic	246	Unlimited-2018	Systematic mapping study
This study	Knowledge evolution trajectory of PHD, including EHR, PHR, and EMR	8281	2009-2018	Bibliometric method

^a^PHR: personal health record.

^b^EHR: electronic health record.

^c^PHD: personal health data.

^d^EMR: electronic medical record.

**Table 2:** Mapping questions.

Question and ID		Mapping question	Rationale
**MQ1^a^: References**			
	MQ1.1	How does the references co-citation network shape?	To understand the main topics and the development of research topics in PHD.^b^
	MQ1.2	How has the knowledge cluster evolved?	To identify which PHD topic has the most longevity and the newest hotspot.
	MQ1.3	What are the citation bursts of reference networks?	To explore the emerging PHD research topic characterized by articles.
**MQ2: Keywords**			
	MQ2.1	What are the keyword bursts in recent years?	To explore the emerging research interests in PHD characterized by keywords.
**MQ3: Disciplines**			
	MQ3.1	What does the discipline categories co-occurrence network shape?	To identify the trends of discipline categories that are involved in PHD.
	MQ3.2	What are the discipline categories bursts?	To explore the discipline categories that increased abruptly in PHD.

^a^MQ: mapping question.

^b^PHD: personal health data.

## Methods

### Data Collection

In 2009, the American Health Information Management Association launched a foundation program “Better health information for all” [2]. From then on, PHD research has developed greatly. Therefore, the time span for the retrieval is from 2009 to 2018 (The data collection was on March 8, 2019). In this review, we relied on scholarly publications in the Web of Science Core Collection, which covers over 21,000 science and social science journals and gives access to multiple databases that reference cross-disciplinary research. Web of Science has been long recognized as an ideal data source for bibliometric analysis.

To ensure the quality of the data set, we retrieved both original research articles and review articles from Science Citation Index Expanded and Social Science Citation Index. As there is no common definition for PHD, the following terms were searched in titles, abstracts, or keywords to identify PHD-related research in the Web of Science database: “personal health data”, “personal health record”, “electronic health record” or “electronic medical record”. In Web of Science, the “Topic Search” function returns results in titles, abstracts, or keywords. Thus, the search query was defined as follows:

TS(Topic)=(“personal health data” OR “personal health record” OR “electronic health record” OR “electronic medical record”) AND DT(Document Types)=(“Articles” OR “Review”) AND PY(Year Published)=(2009-2018).

**Figure 2:**
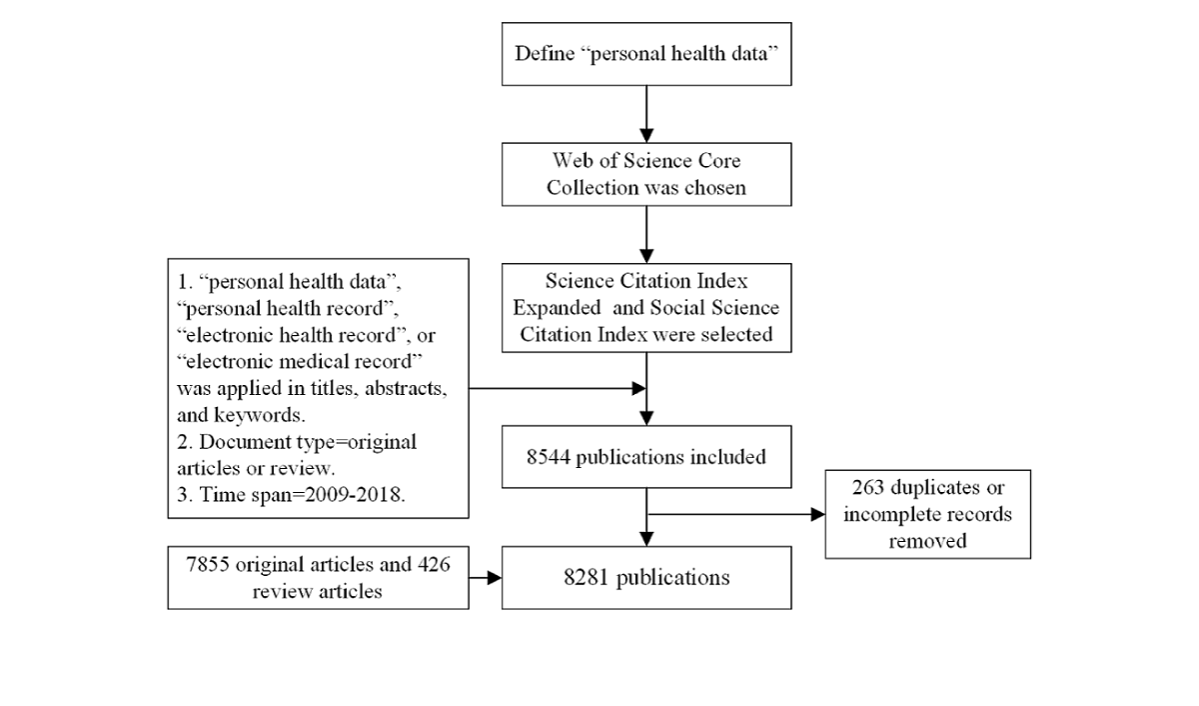
PRISMA (Preferred Reporting Items for Systematic Reviews and Meta-Analyses) flow chart of data selection.

This search yielded 8544 publications. After eliminating publications with replicated or incomplete retrieval data, 8281 records were left, 7855 (94.86%) of which were original articles and 426 (5.14%) review articles. The data set selection process follows the PRISMA (Preferred Reporting Items for Systematic Reviews and Meta-Analyses) flow (Figure 2).

### Data Analysis

#### Overview

**Figure 3:**
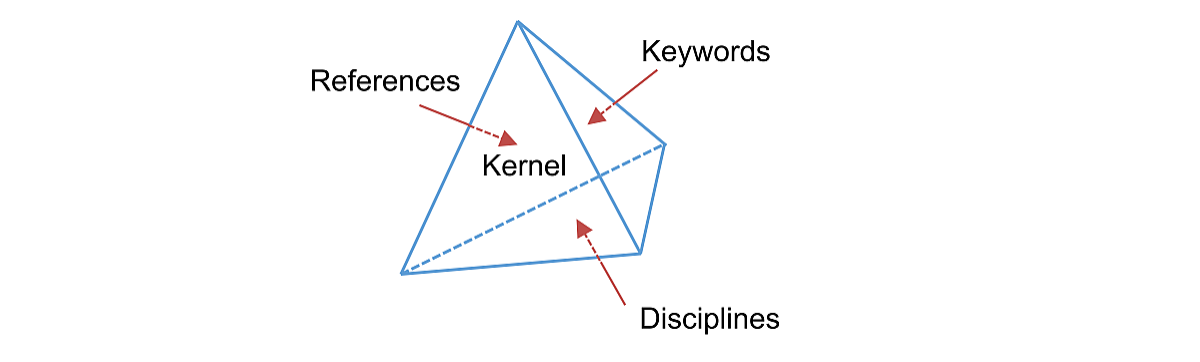
3D attributions of knowledge kernel.

We used KEA to analyze the evolution of PHD research. The KEA followed a bibliometric approach, whereby each article is viewed as a knowledge resource. The relationships of various knowledge resources represent knowledge networks: reference co-citation, keyword co-occurrence, and discipline co-occurrence. These knowledge networks can be analyzed along the 3 dimensions of references, disciplines, and keywords using similarity-based clustering [31,32]. This combination of reference, keyword, and discipline networks represents a knowledge kernel, which is a three-dimensional space depicting the overall knowledge network of a research field (Figure 3). As such, the 3 knowledge networks present the evolution of a knowledge kernel along the 3 dimensions of references, disciplines, and keywords. Taken together, the 3 knowledge networks represent the knowledge evolution of a knowledge kernel. This approach is referred to as KEA. Besides, the burst detection technique was employed to identify emerging research hotspots.

An article typically cites and is cited by many others. To identify the interrelationships between articles, reference co-citation analysis is commonly used. Co-citation analysis can only categorize part of the cited literature in a research field, so keyword co-occurrence and discipline co-occurrence techniques were also used to reveal information on other key topics. These 3 techniques can help analyze the dynamics of a research field over time and are discussed in detail below.

#### Reference Co-citation Network

Small [33] defined co-citation as “the frequency with which two items of earlier literature are cited together by the later literature”. The reference co-citation network was generated with a threshold of 4 or more co-citations [34], and the networks were divided into several clusters, with each network being labeled by terms extracted from the titles of the most representative citing articles [35]. This analysis shows how PHD research focus changes over time.

#### Keywords Co-occurrence Network

A list of predefined keywords represents the core idea of an article. Keyword co-occurrence refers to the statistical correlation between keywords that appear in the same article. A keyword co-occurrence network links keywords listed in the same article and presents the relationships between these keywords as a network map. The shortest distance between any 2 keywords that are not linked directly is viewed as the closeness of the 2 words [34]. The cluster formed by closely linked keywords represents a key subject domain of a research field. The burst detection algorithm shows how keywords emerge through frequency analysis to signify the most active PHD research hotspots over time [36].

#### Disciplines Co-occurrence Network

In this technique, each scientific article is assigned to 1 or more disciplines to calculate the statistical correlation between disciplines. When an article is assigned to 2 disciplines, these disciplines are related, and related disciplines combine to form a discipline co-occurrence network [37]. A burst detection algorithm can be used to detect the most active disciplines in PHD articles [36,38].

In this study, we used CiteSpace 5.2.R2, a bibliometric tool to analyze PHD articles [39].

## Results

In the following sections, we present the KEA of references, disciplines, and keywords in the published PHD research.

### Reference Co-citation Network

We constructed a co-citation network of the top 100 most cited articles each year from 2009 to 2018. Clustering was performed using the log-likelihood ratio method. The analysis identified 15 major clusters. Silhouette values ≥0.7 indicate high similarity among articles in the same cluster, while modularity Q values ≥0.6662 indicate high differences between clusters [34].

**Figure 4:**
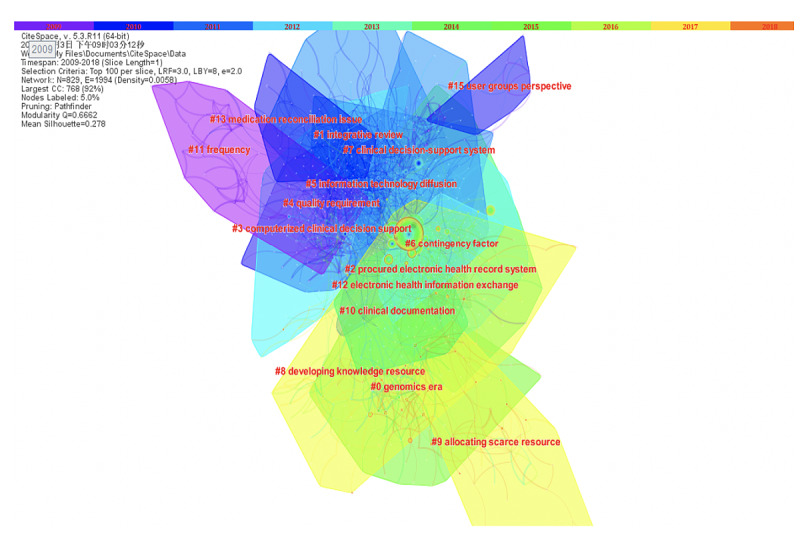
Co-citation clusters of references (Modularity Q=0.6662, Mean Sihouette=0.278, Selection Criteria=Top 100 per slice).

Figure 4 shows the evolution trajectory of the PHD knowledge kernel based on the reference co-citation network. The colored bars at the top of the figure represent different years. The corresponding colored curves represent co-citations occurring in that year. The size of a node depicted with the citation “tree rings” represents the number of times an article was cited [34]. The networks are further decomposed into clusters as tightly coupled references. Each cluster is labeled using terms extracted from noun phrases in titles.

From Figure 4, we can see that the most popular PHD research topics changed over time. Before 2013, knowledge clusters such as clusters 3 (clinical decision support), 5 (information technology diffusion), and 2 (EHR system) mainly focused on medicine and technology. From 2013 onward, the focus shifted to health care resource allocation, such as clusters 8 and 9, focusing on developing knowledge resources and allocating scarce resources. A closer examination of clusters 8 and 9 can be found in Multimedia Appendix 2. It lists articles with coverage ≥9%, which represents the percentage of members in each cluster that articles cite. To some extent, these articles are the most representative articles of each cluster. For example, the articles focusing on developing knowledge resources for precision medicine [40], use of EHRs for clinical decision [41,42], and review of an integrated clinical decision support system [43] are the most representative articles of cluster 8 (developing knowledge resource). Likewise, the articles focusing on scarce resource allocating for heart disease [44], a population-level EHR cohort study [45], and data science application in critical care [46] are the most representative articles of cluster 9 (allocating scarce resource). The major clusters are described in detail in Table 3.

**Table 3:** Description of co-citation clusters.^a^

Mean year^b^	Cluster ID	Size^c^	Silhouette	Label (LLR^d^)
2003	15	10	0.984	User groups perspective
2005	3	69	0.805	Clinical decision support
2005	5	62	0.815	Information technology diffusion
2005	10	31	0.777	Clinical documentation
2006	1	72	0.872	Integrative review
2006	13	18	0.947	Medication reconciliation issue
2007	4	64	0.838	Quality requirement
2007	6	61	0.809	Contingency factor
2010	2	70	0.804	EHR^e^ systems
2010	7	61	0.847	Clinical decision support system
2010	12	30	0.833	Electronic health information exchange
2011	0	95	0.849	Genomic era
2011	11	31	0.936	Frequency
2013	8	51	0.893	Developing knowledge resource
2013	9	43	0.950	Allocating scarce resource

^a^The connected components in cluster 14 are less than the default value (K=25), so CiteSpace did not report 14 [39].

^b^The average year of the articles in a cluster.

^c^The number of articles in each cluster.

^d^LLR: log-likelihood ratio.

^e^EHR: electronic health record.

### Keyword Co-occurrence Network

Multimedia Appendix 3 shows the keyword co-occurrence networks. Multimedia Appendix 4 shows the 56 keywords with the strongest burst out of 100 keywords that were frequently cited each year between 2009 and 2018. This was performed using the “burst detection” function in CiteSpace. In 2009, keywords with the strongest burst mainly focused on basic PHD issues (eg, privacy, physician order entry, and standard) and medical issues (eg, diabetes mellitus, heart disease, blood pressure). Between 2010 and 2013, the keywords clinical information system, database, ambulatory care, personal health record had the strongest burst. Since 2013, burst keywords included attitude and satisfaction, implying that PHD research evolved from focusing on technology- and medicine-centered perspectives to focusing on human-centered perspectives. The most recent burst keywords (eg, readmission, emergency department, usability) appear to be likely PHD research hotspots, focusing on efficiency and quality of health care resources.

### Discipline Co-occurrence Network

**Figure 5:**
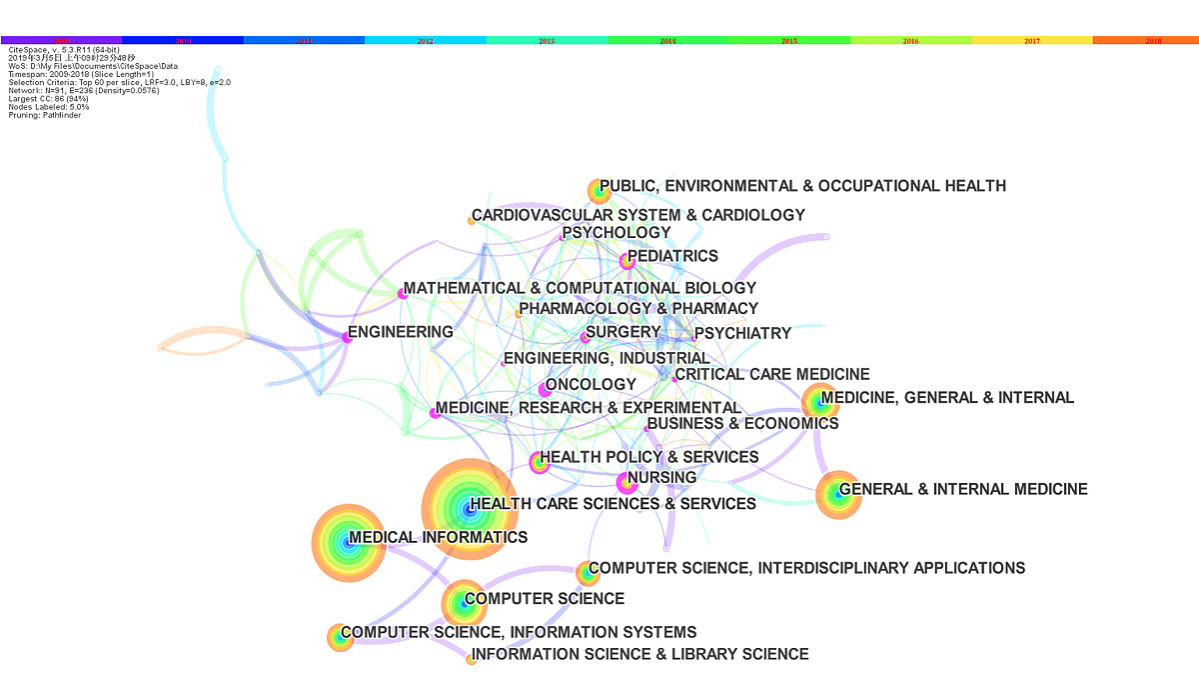
Disciplines co-occurrence network (2009–2018) (Pruning=Pathfinder, Node=91, Density=0.0576, Selection Criteria= Top 60 per slice).

Figure 5 shows the evolution trajectory of the PHD knowledge kernel based on discipline co-occurrence networks. The size of a node represents the number of articles in a specific discipline. The links between nodes show interdisciplinary collaborations. The colors of links show when a connection was made for the first time. The tree rings represent the co-occurrence history of a discipline. The color of a circle ring denotes the time of corresponding citations. The largest node was health care sciences, followed by medical informatics, general and internal medicine, and computer science, indicating that these are the mainstream disciplines in PHD studies. Nodes with high betweenness centrality (indicated by the purple rim) [35], including health policy and services, psychology, and business and economics, may be pivotal to the paradigm shift of PHD research.

Disciplines with the strongest burst are shown in Multimedia Appendix 5. Management was at the top of the list with a burst strength of 4.4358 between 2009 and 2011. Before 2013, most research hotspots, such as biochemistry and molecular biology, dentistry, and oral surgery and medicine, were medicine and biology disciplines. From 2013 to 2016, various technologies were combined into PHD research, including computer science (artificial intelligence [AI]) and medical laboratory technology. Since 2016, substance abuse and psychology disciplines have become more popular in PHD research. Psychology had a relatively high burst strength (6.5215) and appears to be a significant discipline for future research. Social sciences also had a strong burst (4.8105) for the longest time, making it a central focus of PHD research.

## Discussion

### Principal Findings

To the best of our knowledge, this is the first systematic review to show how PHD research has evolved and which research areas are potential hotspots. We examined the PHD knowledge kernel in 3 networks—reference co-citation, keyword co-occurrence, and discipline co-occurrence—to unveil how knowledge clusters evolved, which subjects are key, and which disciplines are being studied in PHD research. The proposed KEA framework can be extended to other similar interdisciplinary research areas. This is also the first study to focus on all types of PHD, including EMR, EHR, and PHR; previous reviews have focused on 1 type of health data. Lastly, this study included a large number of articles (8281 articles) and was not restricted to specific research questions or research types.

The reference co-citation network revealed that PHD research mainly focused on medicine and technology issues (eg, clinical decision systems) before 2013. From 2013 onward, the focus shifted toward developing knowledge resources and allocating scarce health care resources. The results also suggest that from 2013 onward, research communities have been actively seeking methods to make meaningful use of PHD. The overall trend of EHR research mirrors the previous finding of Qian et al [29] that EHR research has evolved from the adoption of EHR to higher-level application and integration of EHR. A well-cited publication is one from Blumenthal and Tavenner [47], which briefs about how EHR benefits patients and caregivers. Other studies have explored the benefits of clinical decision support systems based on EHR as well as barriers to using EHR [18,48,49]. Moreover, the application of PHD in medical research has evolved with technological development. At first, EHR-based clinical decision support systems were mainly used to diagnose and treat specific diseases such as diabetes and heart disease [50]. Later on, more effort was made to develop and systematically incorporate health care data to improve genomics and precision medicine [40].

The reference co-citation network also showed that the most active PHD knowledge cluster is developing knowledge resources and allocating scarce resources. This is supported by the analysis of the keywords that shows PHD studies focusing on emergency health care typically involve the application of the latest knowledge and use of scarce resources [44,46]. The co-citation analysis also demonstrated that the focus of PHD research is moving away from improving treatment decisions to optimizing resource distribution to different groups. This pertains to the allocative value of value-based health care, which aims to equalize resource allocation and improve health care outcomes between different groups [51], thereby improving health care services. In line with the aforementioned, AI applications have proven to be effective, especially in image interpretation [52,53] and diagnosis [54,55]. During the COVID-19 pandemic, the AI system played an important role in rapid early detection and diagnosis [56,57]. AI also can help in optimizing treatment regimens, prevention strategies, and allocation of scarce health resources to narrow down the inequality in health care, especially in resource-poor settings attributed to the shortage of human resources and medical devices [58]. These findings suggest that it is necessary to improve the equity in health resource allocation. Notably, value-based health care and AI applications should be given more attention.

The keyword co-occurrence analysis revealed that technical issues such as data privacy, data standardization, data quality, and interoperability between different information systems were studied first, which makes sense as these are initial and critical steps for using PHD. Data quality is important because it ensures the accuracy of the information provided. Interoperability between information systems is also important for information exchange. Privacy protection encourages people to share their health data. The importance of these technical issues has been well supported by other systematic reviews [6,16,59,60]. These findings suggest that adequate processes for collecting PHD are prerequisites for the utilization of PHD and more effort should be put in place at the initial stage of data standardization and optimizing interoperability.

The bursts in topics related to psychology and human factors (eg, attitude, satisfaction, education) indicate the switch from technology-centric issues to more human-centric issues in PHD studies. The study by Blumenthal [4] and Meier [61] showed that meaningful use of PHD requires more attention to education, attitude, and satisfaction of all the stakeholders. Patient satisfaction is critical for successful health care and depends on quality, communication, and interpersonal interactions with health care providers [62]. Moreover, as AI-based technology including machine learning, natural language processing, and artificial networks is integrated into health care more deeply, the “black box” algorithms have raised concerns about technology liability as well as patient and clinician trust [57,63]. Further research on regulatory issues and governance of PHD is therefore recommended.

Our findings also supported the unified theory of acceptance and use of technology [64], which comprises 4 key elements (ie, performance expectancy, effort expectancy, social influence, and facilitating conditions) that influence how we use technology. These elements are related to how humans interact with technology and make sure that technology creates value for patients, physicians, and administrators, which eventually improves satisfaction. As technologies (eg, AI, internet of things) are now widely used in health care, these issues are gaining more importance [65]. The aforementioned human factors reflect the notion of “personalized value,” another dimension of value-based health care, which emphasizes that every patient should be fully informed about the benefits and risks of treatments [66]. Therefore, the technology developer and health care institutions need to consider these human factors for the effective adoption of PHD-related technology.

The discipline co-occurrence analysis revealed the evolution of PHD research over various disciplines over the past 10 years with a more recent focus on computer science, including AI, machine learning, and deep learning. This agrees with the notion that computer science can increase the value of PHD [11,67]. Yin et al [11] reviewed the effectiveness of machine learning technology in personal health investigations based on online PHD [11], and Payrovnaziri et al [68] conducted a review of AI models that use EHR data. Hou et al [36] pointed out that AI could be used not only as a screening tool to interpret radiology images but also to interpret these images with greater consistency than humans can. Moreover, AI-based technology has the potential to improve efforts toward precision medicine. Tran et al [69] stated that AI technology leverages individual health data and data science to enhance prognosis, diagnosis, and rehabilitation. Regardless of the specific technique or function, the general aim of these technologies is to ease the shortage of human and device resources and optimize the allocation of scarce health care resources. This notion of effective technology application within PHD research presents another dimension of value-based health care known as “technical value” [70]. These findings suggest that all stakeholders should be educated about AI technology to promote value generation through PHD.

Overall, our results indicate that health data analytics should go beyond improving decision-making processes to providing better results for populations [71]. In line with this, PHD research is transitioning toward a more human-centric approach with a new focus on value-based health care: “allocative value,” “technology value,” and “personalized value” [70]. These findings indicate that PHD research has the potential to meet the triple aims of value-based health care in the future.

### Limitations

There are some limitations to this review. First, the scope of the data is limited by the source (the Web of Science) and the search items used. This study did not use “sentiments,” “emotions,” and “social media data” for data set search, as they are not well-defined terminologies or keywords, which might bias the data set. An iterative query refinement would improve the quality of the data set, although the search strategy adequately met the study purpose. Second, the results present an overview of how structure and knowledge have evolved in PHD research; however, details on more specific research topics are lacking. Researchers need to explore this in detail using additional methods and other scholarly publications. Topics to address include health care inequity and cost-effective health care through joint efforts of professional health care networks and patient networks [72]. Third, the co-citation networks rely on citation relationships between articles. While some citations reflect a strong connectedness, other citations might reflect a weaker connectedness. Further research is needed to distinguish between different kinds of citations.

### Conclusions

This study used KEA to review the evolution of PHD research and identify research hotspots. The results show that the focus of PHD research has evolved from medicine centric to technology centric, to human centric since 2009. PHD is applied to optimize the allocation of scarce health care resources and to improve the quality and efficiency of health care services. Moreover, AI-based technology is becoming more relevant in PHD research, and that this technology may be used to ease the shortage of human and device resources. Furthermore, PHD research is now paying more attention to topics related to psychology and human factors, such as education, attitude, and satisfaction of stakeholders. These findings indicate opportunities for interdisciplinary cooperation in several PHD research areas: (1) AI applications for PHD; (2) regulatory issues and governance of PHD; (3) education of all stakeholders about AI technology; (4) value-based health care including “allocative value,” “technology value,” and “personalized value.”

## Appendix

Multimedia Appendix 1 The annual number of published articles on personal health data in Web of Science (2009–2018).

Multimedia Appendix 2 A list of articles that contributed to clusters #8 and #9.

Multimedia Appendix 3 Keywords co-occurrence network.

Multimedia Appendix 4 The 56 keywords with the strongest burst (2009-2018).

Multimedia Appendix 5 The 15 disciplines with the strongest burst (2009–2018).

## Abbreviations

AI: artificial intelligence
EHR: electronic health record
EMR: electronic medical record
KEA: knowledge evolution analysis
PHD: personal health data
PHR: personal health record
PRISMA: Preferred Reporting Items for Systematic Reviews and Meta-Analyses
