# Supplementary figures and images for: Visualizing Knowledge Evolution Trends and Research Hotspots of Personal Health Data Research: Bibliometric Analysis
Source: JMIR Med Inform. 2021 Nov 1;9(11):e31142. doi: 10.2196/31142 (PMC8593818; doi:10.2196/31142)

## Multimedia Appendix 1：The annual number of published articles on PHD in WoS (2009–2018).


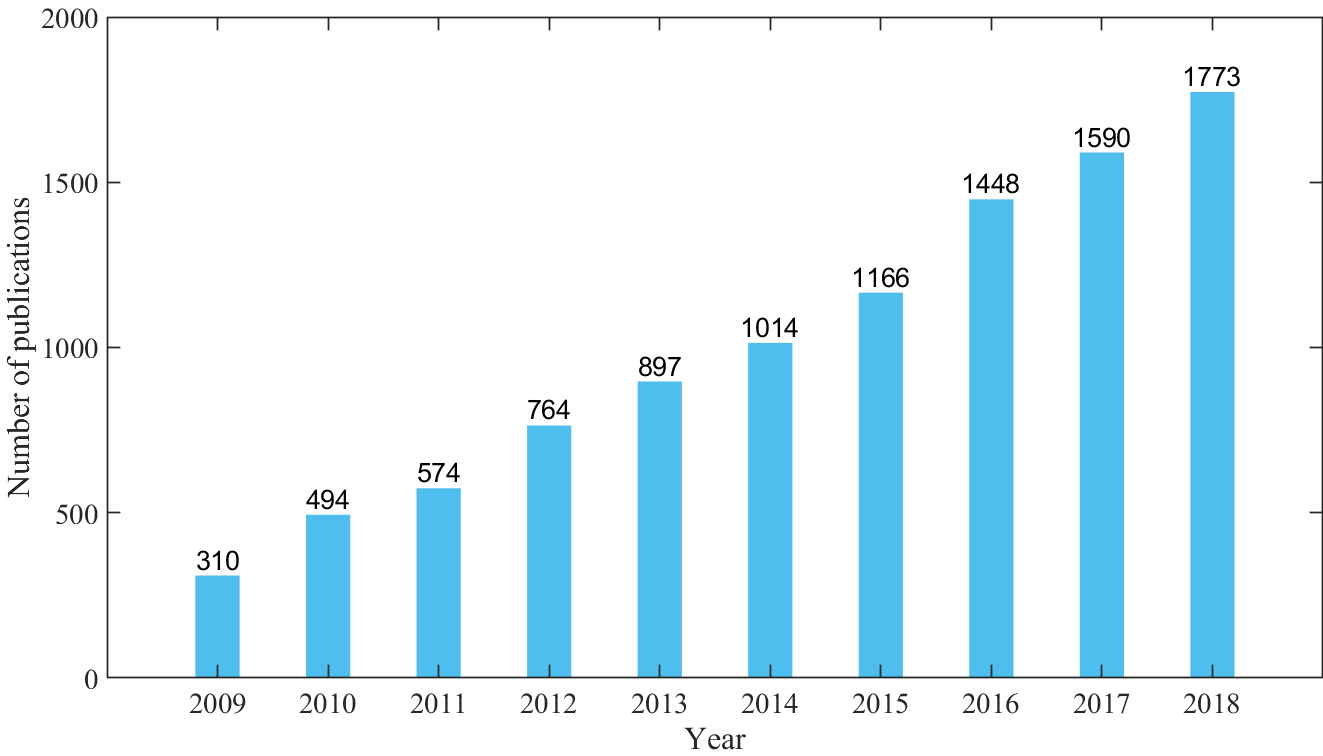

Supplement: Multimedia Appendix 1 [file medinform_v9i11e31142_app1.docx]

## Multimedia Appendix 3: Keywords co-occurrence network.


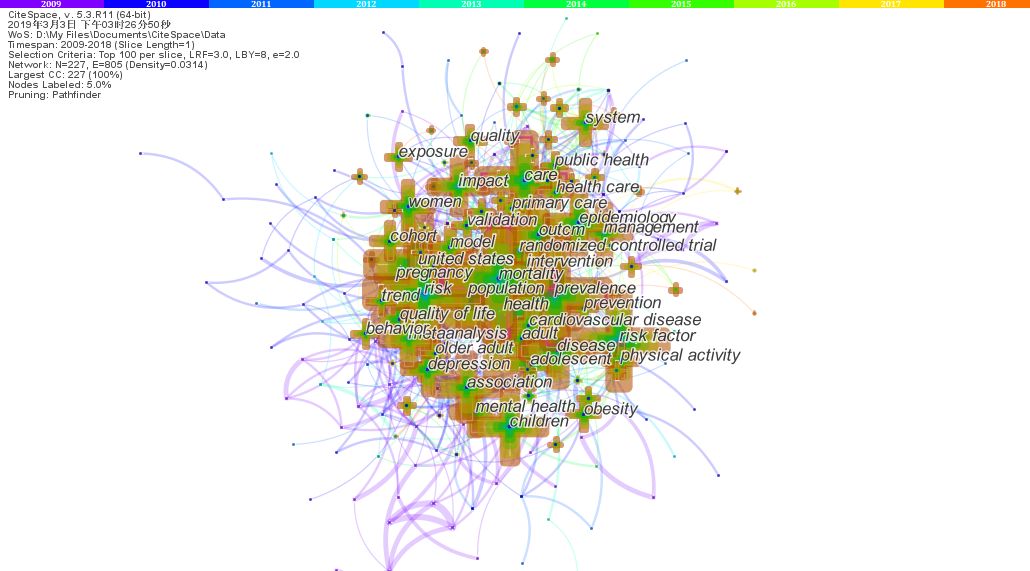

Supplement: Multimedia Appendix 3 [file medinform_v9i11e31142_app3.docx]
