# Supplementary material for: Visualizing Knowledge Evolution Trends and Research Hotspots of Personal Health Data Research: Bibliometric Analysis
Source: JMIR Med Inform. 2021 Nov 1;9(11):e31142. doi: 10.2196/31142 (PMC8593818; doi:10.2196/31142)
Supplement: Multimedia Appendix 2 [file medinform_v9i11e31142_app2.docx]

## Multimedia Appendix 2：A list of articles that contributed to cluster #8 and #9.

| **Cluster #** | **Rank** | **Coverage(%)** | **Representative article** |
| --- | --- | --- | --- |
| 8 | 1 | 18 | Hoffman et al. (2016). Developing knowledge resources to support precision medicine: principles from the Clinical Pharmacogenetics Implementation Consortium (CPIC). |
| 8 | 2 | 16 | Hicks, J. K. et al. (2016). Integrating pharmacogenomics into electronic health records with clinical decision support. |
| 8 | 3 | 14 | Caraballo, P. J. et al. (2017). Electronic Medical Record‐Integrated Pharmacogenomics and Related Clinical Decision Support Concepts. |
| 8 | 4 | 14 | Hinderer, M. et al. (2017). Integrating clinical decision support systems for pharmacogenomic testing into clinical routine-a scoping review of designs of user-system interactions in recent system development. |
| 8 | 5 | 14 | Moyer, A. M., & Caraballo, P. J. (2017). The challenges of implementing pharmacogenomic testing in the clinic. |
| 8 | 6 | 12 | Danahey, K. et al. (2017). Simplifying the use of pharmacogenomics in clinical practice: building the genomic prescribing system. |
| 8 | 7 | 12 | Klein M E, Parvez M M, Shin J G. Clinical implementation of pharmacogenomics for personalized precision medicine: barriers and solutions[J]. |
| 8 | 8 | 12 | Roden, D. M., & Denny, J. C. (2016). Integrating electronic health record genotype and phenotype datasets to transform patient care. |
| 8 | 9 | 10 | Caudle K.E. et al. (2017). Standardizing terms for clinical pharmacogenetic test results: consensus terms from the Clinical Pharmacogenetics Implementation Consortium (CPIC). |
| 8 | 10 | 10 | Rasmussen, L. V., Overby, C. L., Connolly, J., Chute, C. G., Denny, J. C., Freimuth, R. R., ... & Peissig, P. L. (2016). Practical considerations for implementing genomic information resources. Applied clinical informatics, 7(03), 870-882. |
| 9 | 1 | 12 | Amarasingham, R. et al. (2013). Allocating scarce resources in real-time to reduce heart failure readmissions: a prospective, controlled study. BMJ Qual Saf, 22(12), 998-1005. |
| 9 | 2 | 9 | Koudstaal, S. et al. (2017). Prognostic burden of heart failure recorded in primary care, acute hospital admissions, or both: a population‐based linked electronic health record cohort study in 2.1 million people. |
| 9 | 3 | 9 | \| Sanchez-Pinto et al. (2018). Big data and data science in critical care. \| \| --- \| |
| 9 | 4 | 9 | Tabak, Ying P (2013) Development and validation of a mortality risk-adjustment model for patients hospitalized for exacerbations of chronic obstructive pulmonary disease. |
| 9 | 5 | 9 | Xiao, Cao (2018) Opportunities and challenges in developing deep learning models using electronic health records data: a systematic review. |
