# Supplementary material for: Visualizing Knowledge Evolution Trends and Research Hotspots of Personal Health Data Research: Bibliometric Analysis
Source: JMIR Med Inform. 2021 Nov 1;9(11):e31142. doi: 10.2196/31142 (PMC8593818; doi:10.2196/31142)
Supplement: Multimedia Appendix 4 [file medinform_v9i11e31142_app4.docx]

| **Keywords** | **Year** | **Strength** | **Begin** | **End** | **2009–2018** |
| --- | --- | --- | --- | --- | --- |
| internet | 2009 | 7.5353 | **2009** | 2012 | ▃▃▃▃▂▂▂▂▂▂ |
| privacy | 2009 | 5.6889 | **2009** | 2011 | ▃▃▃▂▂▂▂▂▂▂ |
| pattern | 2009 | 4.8309 | **2009** | 2010 | ▃▃▂▂▂▂▂▂▂▂ |
| evaluation | 2009 | 4.8309 | **2009** | 2010 | ▃▃▂▂▂▂▂▂▂▂ |
| computer | 2009 | 10.5003 | **2009** | 2010 | ▃▃▂▂▂▂▂▂▂▂ |
| diabetes | 2009 | 6.7662 | **2009** | 2012 | ▃▃▃▃▂▂▂▂▂▂ |
| computerized | 2009 | 8.7136 | **2009** | 2011 | ▃▃▃▂▂▂▂▂▂▂ |
| adverse drug event | 2009 | 13.5708 | **2009** | 2014 | ▃▃▃▃▃▃▂▂▂▂ |
| glycemic control | 2009 | 6.1215 | **2009** | 2012 | ▃▃▃▃▂▂▂▂▂▂ |
| physician order entry | 2009 | 13.2531 | **2009** | 2014 | ▃▃▃▃▃▃▂▂▂▂ |
| diabetes mellitus | 2009 | 4.5986 | **2009** | 2011 | ▃▃▃▂▂▂▂▂▂▂ |
| EMR ^b^ | 2009 | 7.0004 | **2009** | 2012 | ▃▃▃▃▂▂▂▂▂▂ |
| heart disease | 2009 | 4.8996 | **2009** | 2011 | ▃▃▃▂▂▂▂▂▂▂ |
| general practice | 2009 | 7.4755 | **2009** | 2010 | ▃▃▂▂▂▂▂▂▂▂ |
| controlled trial | 2009 | 5.8378 | **2009** | 2011 | ▃▃▃▂▂▂▂▂▂▂ |
| information system | 2009 | 13.0035 | **2009** | 2014 | ▃▃▃▃▃▃▂▂▂▂ |
| standard | 2009 | 11.3077 | **2009** | 2012 | ▃▃▃▃▂▂▂▂▂▂ |
| of care | 2009 | 8.7136 | **2009** | 2011 | ▃▃▃▂▂▂▂▂▂▂ |
| electronic medical record | 2009 | 7.6236 | **2009** | 2011 | ▃▃▃▂▂▂▂▂▂▂ |
| medication error | 2009 | 8.7136 | **2009** | 2011 | ▃▃▃▂▂▂▂▂▂▂ |
| error | 2009 | 6.9078 | **2009** | 2011 | ▃▃▃▂▂▂▂▂▂▂ |
| administrative data | 2009 | 5.391 | **2009** | 2011 | ▃▃▃▂▂▂▂▂▂▂ |
| blood pressure | 2009 | 10.2901 | **2009** | 2013 | ▃▃▃▃▃▂▂▂▂▂ |
| mellitus | 2009 | 9.8927 | **2009** | 2012 | ▃▃▃▃▂▂▂▂▂▂ |
| clinical information system | 2009 | 8.7531 | **2010** | 2011 | ▂▃▃▂▂▂▂▂▂▂ |
| alert | 2009 | 6.4176 | **2010** | 2011 | ▂▃▃▂▂▂▂▂▂▂ |
| database | 2009 | 14.1197 | **2010** | 2014 | ▂▃▃▃▃▃▂▂▂▂ |
| hospitalized patient | 2009 | 5.2502 | **2010** | 2011 | ▂▃▃▂▂▂▂▂▂▂ |
| ambulatory care | 2009 | 17.6853 | **2010** | 2012 | ▂▃▃▃▂▂▂▂▂▂ |
| order entry | 2009 | 5.8338 | **2010** | 2011 | ▂▃▃▂▂▂▂▂▂▂ |
| performance | 2009 | 2.9943 | **2010** | 2011 | ▂▃▃▂▂▂▂▂▂▂ |
| framework | 2009 | 5.5085 | **2010** | 2012 | ▂▃▃▃▂▂▂▂▂▂ |
| information technology | 2009 | 3.7294 | **2010** | 2011 | ▂▃▃▂▂▂▂▂▂▂ |
| unintended consequence | 2009 | 5.8338 | **2010** | 2011 | ▂▃▃▂▂▂▂▂▂▂ |
| asthma | 2009 | 3.381 | **2010** | 2011 | ▂▃▃▂▂▂▂▂▂▂ |
| personal health record | 2009 | 10.7112 | **2010** | 2012 | ▂▃▃▃▂▂▂▂▂▂ |
| security | 2009 | 6.7178 | **2010** | 2013 | ▂▃▃▃▃▂▂▂▂▂ |
| patient outcome | 2009 | 8.6692 | **2010** | 2012 | ▂▃▃▃▂▂▂▂▂▂ |
| perception | 2009 | 6.663 | **2012** | 2014 | ▂▂▂▃▃▃▂▂▂▂ |
| breast cancer | 2009 | 6.9076 | **2012** | 2014 | ▂▂▂▃▃▃▂▂▂▂ |
| immunization | 2009 | 9.5416 | **2012** | 2013 | ▂▂▂▃▃▂▂▂▂▂ |
| design | 2009 | 10.2192 | **2013** | 2014 | ▂▂▂▂▃▃▂▂▂▂ |
| decision support system | 2009 | 7.2158 | **2013** | 2014 | ▂▂▂▂▃▃▂▂▂▂ |
| efficiency | 2009 | 9.3826 | **2013** | 2014 | ▂▂▂▂▃▃▂▂▂▂ |
| survival | 2009 | 6.521 | **2013** | 2014 | ▂▂▂▂▃▃▂▂▂▂ |
| nurse | 2009 | 5.6294 | **2014** | 2015 | ▂▂▂▂▂▃▃▂▂▂ |
| barrier | 2009 | 4.7174 | **2014** | 2015 | ▂▂▂▂▂▃▃▂▂▂ |
| attitude | 2009 | 13.6387 | **2014** | 2016 | ▂▂▂▂▂▃▃▃▂▂ |
| event | 2009 | 9.3717 | **2015** | 2016 | ▂▂▂▂▂▂▃▃▂▂ |
| education | 2009 | 8.1489 | **2015** | 2018 | ▂▂▂▂▂▂▃▃▃▃ |
| satisfaction | 2009 | 7.3447 | **2015** | 2016 | ▂▂▂▂▂▂▃▃▂▂ |
| challenge | 2009 | 9.986 | **2016** | 2018 | ▂▂▂▂▂▂▂▃▃▃ |
| usability | 2009 | 10.4113 | **2016** | 2018 | ▂▂▂▂▂▂▂▃▃▃ |
| rate | 2009 | 12.6774 | **2016** | 2018 | ▂▂▂▂▂▂▂▃▃▃ |
| readmission | 2009 | 9.4994 | **2016** | 2018 | ▂▂▂▂▂▂▂▃▃▃ |
| emergency department | 2009 | 8.4423 | **2016** | 2018 | ▂▂▂▂▂▂▂▃▃▃ |
