# Supplementary material for: Visualizing Knowledge Evolution Trends and Research Hotspots of Personal Health Data Research: Bibliometric Analysis
Source: JMIR Med Inform. 2021 Nov 1;9(11):e31142. doi: 10.2196/31142 (PMC8593818; doi:10.2196/31142)
Supplement: Multimedia Appendix 5 [file medinform_v9i11e31142_app5.docx]

| **Discipline Categories** | **Year** | **Strength** | **Begin** | **End** | **2009 - 2018** |
| --- | --- | --- | --- | --- | --- |
| Management | 2009 | 4.4358 | **2009** | 2011 | ▃▃▃▂▂▂▂▂▂▂ |
| Social Sciences-Other Topics | 2009 | 4.8105 | **2009** | 2014 | ▃▃▃▃▃▃▂▂▂▂ |
| Ethics | 2009 | 2.4464 | **2009** | 2010 | ▃▃▂▂▂▂▂▂▂▂ |
| Biochemistry & Molecular Biology | 2009 | 2.4352 | **2011** | 2015 | ▂▂▃▃▃▃▃▂▂▂ |
| Toxicology | 2009 | 5.2411 | **2012** | 2013 | ▂▂▂▃▃▂▂▂▂▂ |
| Education, Scientific Disciplines | 2009 | 4.5037 | **2012** | 2015 | ▂▂▂▃▃▃▃▂▂▂ |
| Computer Science, Artificial Intelligent | 2009 | 4.2559 | **2013** | 2016 | ▂▂▂▂▃▃▃▃▂▂ |
| Dentistry, Oral Surgery & Medicine | 2009 | 2.6363 | **2013** | 2018 | ▂▂▂▂▃▃▃▃▃▃ |
| Genetics & Heredity | 2009 | 3.157 | **2013** | 2014 | ▂▂▂▂▃▃▂▂▂▂ |
| Pathology | 2009 | 7.6078 | **2014** | 2015 | ▂▂▂▂▂▃▃▂▂▂ |
| Biotechnology & Applied Microbiology | 2009 | 6.5891 | **2015** | 2016 | ▂▂▂▂▂▂▃▃▂▂ |
| Anesthesiology | 2009 | 3.9976 | **2015** | 2018 | ▂▂▂▂▂▂▃▃▃▃ |
| Medical Laboratory Technology | 2009 | 4.6415 | **2015** | 2016 | ▂▂▂▂▂▂▃▃▂▂ |
| Substance Abuse | 2009 | 3.7304 | **2016** | 2018 | ▂▂▂▂▂▂▂▃▃▃ |
| Psychology, Clinical | 2009 | 6.5215 | **2016** | 2018 | ▂▂▂▂▂▂▂▃▃▃ |
